# Engineering of Family-5 Glycoside Hydrolase (Cel5A) from an Uncultured Bacterium for Efficient Hydrolysis of Cellulosic Substrates

**DOI:** 10.1371/journal.pone.0065727

**Published:** 2013-06-13

**Authors:** Amar A. Telke, Ningning Zhuang, Sunil S. Ghatge, Sook-Hee Lee, Asad Ali Shah, Haji Khan, Youngsoon Um, Hyun-Dong Shin, Young Ryun Chung, Kon Ho Lee, Seon-Won Kim

**Affiliations:** 1 Division of Applied Life Sciences (BK21), PMBBRC, Gyeongsang National University, Jinju, Republic of Korea; 2 Center for Environmental Technology Research, KIST, Seoul, Republic of Korea; 3 School of Chemical and Biomolecular Engineering, Georgia Institute of Technology, Atlanta, Georgia, United States of America; 4 Department of Microbiology, School of Medicine, Gyeongsang National University, Jinju, Republic of Korea; Oak Ridge National Laboratory, United States of America

## Abstract

Cel5A, an endoglucanase, was derived from the metagenomic library of vermicompost. The deduced amino acid sequence of Cel5A shows high sequence homology with family-5 glycoside hydrolases, which contain a single catalytic domain but no distinct cellulose-binding domain. Random mutagenesis and cellulose-binding module (CBM) fusion approaches were successfully applied to obtain properties required for cellulose hydrolysis. After two rounds of error-prone PCR and screening of 3,000 mutants, amino acid substitutions were identified at various positions in thermotolerant mutants. The most heat-tolerant mutant, Cel5A_2R2, showed a 7-fold increase in thermostability. To enhance the affinity and hydrolytic activity of Cel5A on cellulose substrates, the family-6 CBM from *Saccharophagus degradans* was fused to the *C*-terminus of the Cel5A_2R2 mutant using overlap PCR. The Cel5A_2R2-CBM6 fusion protein showed 7-fold higher activity than the native Cel5A on Avicel and filter paper. Cellobiose was a major product obtained from the hydrolysis of cellulosic substrates by the fusion enzyme, which was identified by using thin layer chromatography analysis.

## Introduction

Plant cell walls are important resources for the production of ethanol as a next-generation biofuel. Cellulose, a β-1-4-glucose polymer, is a major polysaccharide present in the plant cell wall. The synergistic action of various cellulases, such as cellobiohydrolase, endoglucanase, cellodextrinase, and β-glucosidase, are required for the production of fermentable sugar from cellulose substrates [1]. The most challenging technological and economical obstacle involves the release of fermentable soluble sugars at prices competitive with those used in breaking down sugarcane and corn kernels [2], [3], which can be achieved by increasing the rate of cellulose hydrolysis using wild-type enzymes and protein engineering. Most cellulases with high hydrolytic efficiency are patented and not freely available to researchers. One approach for obtaining efficient cellulases is isolating novel cellulases from cellulolytic microorganisms or metagenomic libraries of uncultured microorganisms, followed by cellulase engineering to enhance cellulose degradation.

Enhanced thermostability is one of the most desirable properties from the cellulase engineering perspective [4]. Large-scale saccharification reactions are carried out at elevated temperatures; thus, a thermostable enzyme is required to maximize hydrolysis product yield. Thermostable enzymes are generally robust, tolerate various harsh conditions used for cellulosic biomass treatment, and withstand long-term usage or storage. Therefore, enzymes that are both thermostable and highly efficient in the saccharification reaction are necessary. Directed molecular evolution is used to enhance cellulase enzyme activities on soluble cellulose substrates and stability at higher temperature [5], [6]. The influence of cellulose binding modules (CBMs) on catalytic domain stability at higher temperatures has been extensively studied 7,8. Additionally, CBM fusion to the catalytic domain enhances cellulase enzyme activities on insoluble cellulose substrates [9], [10]. In this study, we performed random mutagenesis and CBM protein fusion using a cellulase, Cel5A, derived from a metagenomic library of vermicompost [11] to improve its thermostability and catalytic efficiency on cellulosic substrates.

## Materials and Methods

### Chemicals, Bacterial Strains, and Plasmids

All chemicals were of the highest purity available and of an analytical grade. Avicel PH101, carboxymethyl cellulose (CMC), barley β-glucan, *p-*Nitrophenyl-β-D-cellobioside (pNPC), and *p*-Nitrophenyl-β-D-glucopyranoside (pNPG) were obtained from Sigma (St. Louis, MO). Cellobiose, cellotriose, cellotetraose, cellopentose, and cellohexose were obtained from Megazyme (Wicklow, Ireland). The *Cel5A* gene was isolated from the metagenomic library prepared from vermicompost [11]. The strains, plasmids, and genomic DNAs used in this study are listed in Table S1. Genomic DNA extraction was performed according to the method by Sambrook and Russel [12]. Plasmid DNA was extracted using the QIAGEN spin column plasmid mini-preps kit (Hilden, Germany). Plasmid and PCR products were recovered from agarose gel using the QIAGEN gel extraction kit. All nucleotide primers were obtained from Bioneer Co., Ltd. (Daejeon, South Korea) and are listed in Table S2.

### Analysis of Periplasmic Secretion of Cel5A

The gene sequence of *Cel5A* is deposited in the NCBI gene bank under the accession number JN012243 and contains 363 amino acids with a molecular mass of 41.6 kDa [11]. The secretion signal-peptide sequence of Cel5A was predicted using the SignalP 3.0 server at http://www.cbs.dtu.dk/services/SignalP-3.0/ [13]. The first 25 amino acids of the *N*-terminus were predicted to be the signal peptide belonging to type II secretion-signal sequence family in gram-negative bacteria. To elucidate the role of the predicted signal peptide, we artificially removed the signal sequence from the native *Cel5A*. We constructed two plasmids, pTCel5A containing the full-length *Cel5A* gene and pTw/o-ssCel5A containing the truncated *Cel5A* gene without the signal-peptide sequence (w/o-ss) to evaluate the role of the signal sequence in the *Cel5A* gene. *Escherichia coli* BL21 transformants (1×10^6^ colony-forming units; CFU) harboring recombinant plasmids pTw/o-ssCel5A and pTCel5A, respectively were spotted onto Luria broth (LB)-ampicillin-agar plates and incubated at 37°C for 6 h. Hydrolytic activity of *Cel5A* enzyme was evaluated using the Congo red plate assay. To confirm the presence of Cel5A in periplasmic space, we elucidated the protein profile of periplasmic space by SDS-PAGE. The periplasmic protein fractions were prepared according to method described in pET system manual.

### Construction of Cel5A Error-prone PCR Library

The Diversify PCR Random Mutagenesis Kit (Clontech, Mountain View, CA) was used to construct the *Cel5A* mutant library using error-prone PCR (EP-PCR). Random *Cel5A* mutant libraries were prepared according to the protocol as described by the manufacturer. The pTCel5A-outer-F and pTCel5A-outer-R primers were used for the first round of EP-PCR using pTCel5A as a template. PCR was performed according to manufacturer’s instructions. The resulting PCR product was then treated with *Dpn*I to remove the template plasmid, purified using a gel extraction kit, and then used as the template for nested PCR using *Pfu* DNA polymerase (SolGent, Daejeon, South Korea). The pTCel5A-Inner-F and pTCel5A-Inner-R primers were utilized for nested PCR amplification of mutated gene products. Amplified products were purified using a gel extraction kit, and then digested with restriction enzymes, followed by ligation into the pTrc99A vector. The ligation mixture was used to transform chemically competent *E. coli* DH5α. Transformants were grown overnight at 37°C on LB-ampicillin agar medium. Mutation frequency was confirmed by plasmid DNA sequencing of 10 randomly selected transformants. Plasmid DNAs were then extracted to obtain the mutated gene library for subsequent transformation and screening.

### Screening of Cel5A Thermotolerant Mutants

Two-step screening procedure was applied for screening of thermotolerant mutants from the Cel5A mutant library to evaluate cellulase activity on solid (Congo red plate assay) and in liquid medium (96-well plate containing LB medium). Clones showing the highest hydrolytic activity (evaluated using the Congo red plate assay) at elevated temperatures were selected and used for the next screening step. Selected transformants were grown in 96-deep well plates (Thermo Scientific, Waltham, MA) containing 0.5 mL of LB-ampicillin medium. The 96-deep-well plates were incubated at 37°C under a shaking condition (120 rpm). The cultures were initially induced with 0.1 mM IPTG and then incubated at 37°C for 2 h. Next, 200 µL of the culture supernatant from each well of the 96-deep-well plates was transferred to fresh 96-deep-well plates. CMC (0.5%, w/v) was added to each well, followed by incubation at 65°C for 20 min. The residual enzyme activity was calculated using the 3,5-Dinitrosalicylic acid (DNS) method. An abiotic control with no inoculation was included for each assay. The residual enzyme activity was calculated by using following formula.

Residual enzyme activity (%) = [Enzyme activity (U/mL) at t = 20 min/Enzyme activity (U/mL) at t = 0 min]×100.

Mutants showing high enzyme activity at elevated temperature were selected and subjected to gene sequencing. The most thermotolerant *Cel5A* mutant gene was amplified using Cel5A-F1 and Cel5A-R1 primers, followed by cloning into the expression vector pET-28a(+).

### Computational Analysis of Mutations

Models of mutants were constructed by changing the corresponding amino acids in the crystal structure model of Cel5A in complex with a cellobiose (PDB ID: 4HUO) using COOT [14]. Three-dimensional models of each mutants and wild-type structures were then energy-minimized using the conjugate gradient minimization method in the program CNS [15], [16]. Hydrogen atoms for all amino acids in the models were generated and included for the calculations. The region within 10 Å from the mutated residues was included for minimization while the rest of the model was restrained harmonically. Strong harmonic restraints were applied to keep the backbone relatively fixed during minimization; 500 minimization steps were run for each mutant model. The total energy of each mutant model was calculated as the sum of the energies from bond, angle, torsion, Van der Waals, and electrostatic interactions calculated using CNS. Solvent accessible surface area and buried surface area for atoms were calculated using MS in CCP4 [17]. Molecular structure representations were produced using PyMOL [18].

### Effect of CBM6 on Activity of Thermotolerant Mutant Cel5A_2R2

CBM6 was fused to the *C*-terminus of Cel5A_2R2 using 3-step overlap PCR [19]. The construction procedure is shown in Figure S1. Primers utilized for this study are shown in Table S2. pTCel5A**_**2R2 and pECel5H were used as template DNA for amplifying the full-length mutant Cel5A_2R2 and family-6 CBM, respectively. CBM6 present in the endoglucanase Cel5H from *Saccharophagus degradans* was used for protein fusion because CBM6 was known to enhance the activity of the catalytic domain towards insoluble substrates [20]. Phusion high-fidelity DNA polymerase (Finnzymes, Vantaa, Finland) was utilized in the PCR reaction according to the manufacturer’s instructions.

### Expression, Purification, and SDS-PAGE Analysis of Recombinant Proteins

*E. coli* BL21(DE3) harboring recombinant plasmids were cultured at 37°C in LB medium containing 50 µg/mL kanamycin. The culture were induced with 0.2 mM IPTG, when cell growth was 0.7 at OD_600_ nm, and incubated overnight at 22°C. Cells were harvested by centrifugation at 10,000 rpm for 20 min at 4°C. Recombinant proteins were extracted using the CelLyticB system (Sigma) according to the manufacturer’s instructions and then purified on a pre-charged His-Trap nickel sepharose column from GE Healthcare/Amersham (Piscataway, NJ, USA) under native conditions. Binding was achieved in the presence of 20 mM imidazole followed by washing with 1× sodium phosphate buffer (pH 7.5–8.0) containing 40 mM imidazole. Finally, elution was carried out using 250 mM imidazole. The fractions containing the desired proteins were pooled and dialyzed overnight at 4°C against 50 mM Tris-HCl buffer (pH 8.0). Finally, the enzyme was stored in 50 mM Tris-HCl buffer (pH 8.0) containing 10% glycerol. Expression levels of wild-type and mutant proteins in *E. coli* cells were quantified using the S-Tag Rapid Assay Kit (Novagen, Darmstadt, Germany) according to manufacturer’s instructions. Protein concentration was determined using a BCA protein assay kit (Pierce, Rockford, IL, USA) with purified bovine serum albumin used as the standard. SDS-PAGE was carried out on a vertical polyacrylamide slab gel. Electrophoresis was performed with 5% stacking and 12% polyacrylamide gels under denaturing conditions. Buffers used for the stacking and separating gels were 1 M Tris-HCl (pH 6.8) and 1.5 M Tris-HCl (pH 8.8), respectively.

### Enzyme Activity Assays

Cellulase activity was generally analyzed at pH 5.5–6.0 and 50–55°C in a reaction mixture containing 50 mM sodium acetate or sodium phosphate buffer and 0.5–1.0 nmol purified enzyme. Sodium acetate (pH 4.0 to 5.6) and sodium phosphate (pH 6.0 to 8.0) buffers were used to maintain the desired pH at a final concentration of 50 mM. CMC and barley β-glucan hydrolysis reactions were performed with 1% (w/v) substrate in a total volume of 0.5 mL for 10 min. Phosphoric acid-swollen cellulose (PASC) was prepared based on previous reports [21], [22]. The PASC (0.1%, w/v) hydrolysis reaction was performed in a total volume of 0.5 mL for 10 min. Product formation in these reactions was quantified using the 3,5-Dinitrosalicylic acid (DNS) method [23]. Avicel (1%, w/v) and Whatman no. 1 filter paper (1.5%, w/v) hydrolysis reactions were performed in a total volume of 0.2 mL for 2 h at 55°C. The reactions were stopped by incubation for 10 min at 95°C and residual substrates were separated by centrifugation at 10,000 rpm. The hydrolysis products of Avicel and the filter paper were further digested using 50 U of β-glucosidase (Sigma) in a total volume of 0.35 mL at 37°C for 2 h. The β-glucosidase was inactivated by incubation at 95°C for 10 min. Glucose concentration in the final reaction solution was measured using a glucose oxidase kit (GAGO-20; Sigma) according to the manufacturer’s instructions. Hydrolysis of *p*-nitrophenyl-β-D-glycoside (10 mM) and *p*-nitrophenyl-β-D-cellobioside (10 mM) was assayed by monitoring the concentration of released *p*-nitrophenol at 410 nm after addition of NaOH at a final concentration of 0.1 M [24]. The decrease in viscosity of the CMC solution was measured using a Brookfield DV-III viscometer (Middleboro, MA) at 25°C. Cellulase (0.1 nmol) was added to 0.5% CMC solution and incubated at 55°C followed by viscosity measurements at specified time intervals.

The Congo red plate assay was performed at room temperature (25°C). An LB-ampicillin agar plate containing transformants was overlaid with 0.5% CMC containing top agar (0.75%) followed by induction with 0.5 mM IPTG and incubation at 37°C for 2 h. Plates were flooded with an aqueous solution of Congo red (1%, w/v) with intermittent shaking at 30 rpm for 15 min followed by washing with 1 M sodium chloride. Hydrolytic activity was determined based on the size of the clear halo zone around the bacterial colony.

### Avicel Binding Assay

Avicel binding properties of the native and CBM6 fusion proteins was carried out according to a previous study [25]. The binding assay was carried out in a 2 mL tube. Avicel (5 mg) was mixed with 0.5% bovine serum albumin in sodium phosphate buffer (50 mM, pH 6.0). The mixture was incubated at room temperature for 30 min to avoid nonspecific binding of cellulases. Equal amounts (0.1 nmol) of Cel5A_2R2 and Cel5A_2R2-CBM6 proteins were added to the Avicel solution. Tubes were placed in an Intelli-mixer RM-2 (Rose Scientific Ltd., Edmonton, Alberta, Canada) for 1 h at a rotation speed of 20 rpm. Binding reaction mixtures were centrifuged at 13,000 rpm for 5 min and the amount of unbound enzyme was estimated based on the residual activity in the supernatant. The amount of Avicel-bound enzyme was calculated as the difference between the initial enzyme activity and unbound enzyme activity. Enzyme activity was determined using CMC as a reaction substrate. A control reaction in the absence of Avicel was performed under the same conditions.

### Synergistic Interaction between Cel5A_2R2-CBM6 and CbhA

The synergistic interaction between Cel5A_2R2-CBM6 and cellobiohydrolase A (CbhA) was investigated. The reaction mixture contained 3.5% (w/v) of filter paper disc, 50 mM sodium acetate buffer (pH 5.6), 10 mM CaCl_2_, and equal amounts of CbhA and Cel5A_2R2-CBM6. The reaction was carried out at 55°C for 10 h and the reducing sugar was measured using DNS. The degree of the synergistic effect (DSE) was defined as the ratio of the observed activity of combined enzymes to the sum of observed individual activities.

### Thin-layer Chromatography

Hydrolysis reactions of various cellulosic substrates were stopped by incubating the reaction mixture at an elevated temperature (95°C) for 10 min. The clear supernatant was collected after centrifugation at 1,000 rpm for 20 min and used for further analysis. One-microliter samples were spotted onto Silica gel-60 plates (Sigma), which were air-dried. Thin-layer chromatography (TLC) was developed using a mixture of nitromethane, 1-propanol, and water in a volume ratio of 2∶5:1.5. The TLC plate was dipped into a mixture of 0.3% (w/v) α-naphthol and 5% (v/v) sulfuric acid in methanol and heated to 110°C for 10 min to visualize the resolved products [20], [26].

### Statistical Analysis

All experiments were performed in triplicates and data were analyzed by ‘t’ test. Readings were considered statistically significant when the two-tailed P value is less than 0.0001.

## Results

### Periplasmic Secretion of Cel5A

*E. coli* BL21 harboring pTCel5A showed a clear halo zone on LB agar plates containing 0.5% CMC, whereas no halo zone was observed for *E. coli* BL21 harboring pTw/o-ssCel5A (Figure S2). Thus, the signal-peptide sequence contributes to secretion of Cel5A from *E. coli* BL21 (pTCel5A) containing the full-length Cel5A gene. Extracellular CMCase activity was analyzed to confirm extracellular secretion of Cel5A from *E. coli* BL21 (pTCel5A), but the extracellular activity was found to be below the detection limit. Periplasmic secretion of Cel5A by the signal-peptide sequence was suspected. To determine the periplasmic location of Cel5A, SDS-PAGE was performed using the periplasmic fraction obtained from recombinant *E. coli* (Figure S3). A visible band of Cel5A was observed in the periplasmic fraction of *E. coli* BL21 (pTCel5A), whereas no band was present for the periplasmic fractions of *E. coli* BL21 harboring pTrc99A or pTw/o-ssCel5A. Although Cel5A was not secreted into the extracellular space, a clear halo zone on the CMC-LB agar plate was observed for *E. coli* BL21 (pTCel5A). This indicates slight leakage of the Cel5A protein through the outer membrane into the culture medium. Thus, the signal-peptide sequence is required to transport Cel5A from the cytoplasm into the periplasm.

### Improvement of Cel5A Thermotolerance by Random Mutagenesis

**Figure 1:**
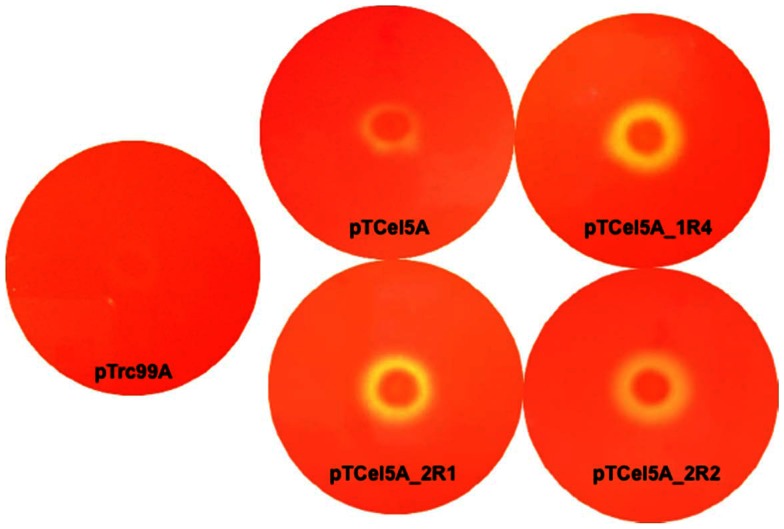
Congo red plate assay for evaluating protein stability at elevated temperature. *E. coli* BL21 transformants (1×10^5^ CFU), harboring pTrc99A, pTCel5A, pTCel5A_1R4, pTCel5A_2R1, and pTCel5A_2R2, were spotted onto LB-ampicillin agar plates, cultivated at 37°C for 6 h, and then induced with 0.5 mM IPTG for 2 h. The plates were overlaid with 0.5% CMC, and incubated at 65°C for 30 min. Finally, plates were stained with Congo red and destained with 1 M sodium chloride.

**Figure 2:**
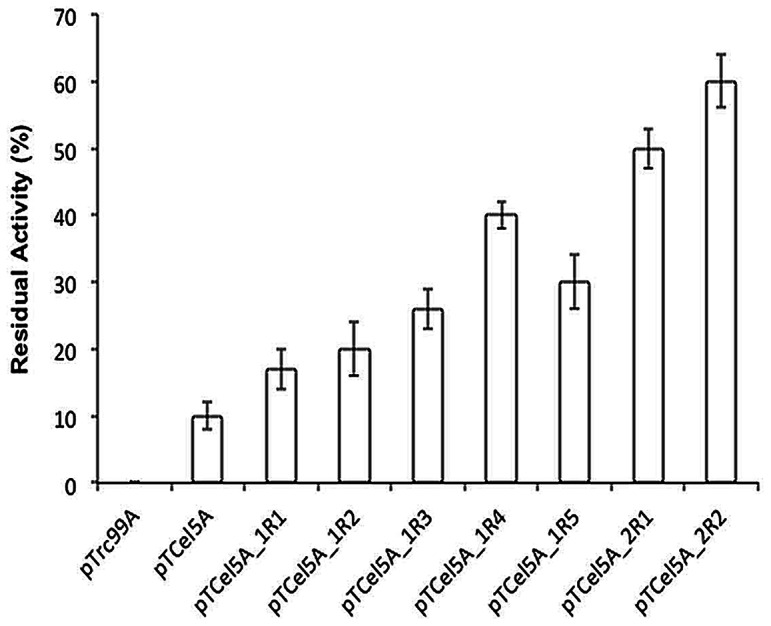
*In vitro* thermotolerance assay for wild-type Cel5A and its mutants. Enzyme was incubated at 65°C for 20 min and residual activity was determined using CMC as a substrate at optimum reaction conditions. Residual enzyme activity (%) = (Enzyme activity (U/mL) at t = 20 min/Enzyme activity (U/mL) at t = 0 min)×100. The error bars represent the standard deviation of triplicate measurements.

Cel5A was unstable at elevated temperature, losing 99% of its enzymatic activity within 30 min of incubation even at 65°C. Despite the importance of cellulases reactions at elevated temperatures, little attention has been given to a conformational stability of these enzymes. Thus, random mutagenesis using EP-PCR was performed to improve Cel5A thermostability. EP-PCR was carried out using the native Cel5A gene containing its secretion-signal sequence as a template. Screening of functional mutants was possible on CMC-LB plates with Congo-red staining because of the extracellular leakage of Cel5A. From the first round of EP-PCR mutation, five thermotolerant mutants, Cel5A_1R1, Cel5A_1R2, Cel5A_1R3, Cel5A_1R4, and Cel5A_1R5, were selected based on the halo zone size on the CMC-LB plates and the residual enzyme activity after a heat treatment at 65°C. The mutated genes were sequenced to identify the mutations responsible for thermal stability. Two or three amino acids were mutated in each mutant (Table 1). Among the thermotolerant mutants, Cel5A_1R4 showed a large halo zone and the highest residual enzyme activity (40% of initial activity) at the elevated temperature (Figure 1 and 2). Thus, Cel5A_1R4 was subjected to a second round of EP-PCR. Two further improved thermotolerant mutants of Cel5A_2R1 and Cel5A_2R2 were obtained from the second round of mutagenesis, which respectively presented 1.25- and 1.5-fold increases in thermostability than the first round mutant Cel5A_1R4. Cel5A_2R1 and Cel5A_2R2 showed a large halo zone size and the residual activities of 50% and 60%, respectively at the elevated temperature (Figure 1 and 2). The size of the halos around the colonies was well-correlated with the observed thermal stability improvement. Cel5A_2R1 and Cel5A_2R2 were purified to homogeneity (Figure S4), followed by further biochemical characterization. Optimum reaction temperature of Cel5A_2R1 and Cel5A_2R2 mutants was increased to 55°C from 50°C of wild-type Cel5A (Table S3). Optimum pH of these mutants was the same as pH5.5 of Cel5A, which was not changed. Thus, specific enzyme activity was measured at 55°C and pH 5.5. Specific activities of Cel5A_2R1 and Cel5A_2R2 were slightly lower than that of Cel5A (Table S3). Expression levels of Cel5A, Cel5A_2R1, and Cel5A_2R2 proteins in *E. coli* BL21 harboring pTCel5A_S-tag, pTCel5A_2R1_S-tag, and pTCel5A_2R2_S-tag plasmids, respectively, were measured using the S-Tag Rapid Assay Kit. Expression levels of Cel5A_2R1 and Cel5A_2R2 were slightly inferior to that of Cel5A. Thus, the thermotolerant mutants Cel5A_2R1 and Cel5A_2R2 were obtained from Cel5A using random mutagenesis with EP-PCR. Cel5A_2R2 was used for further protein engineering studies because it presented higher thermotolerance and specific activity than Cel5A_2R1.

**Table 1:** Mutations obtained after random mutagenesis.

Mutation rounds	Mutants	Substitutions of amino acids and nucleotides*
1st	1R1	D45G (A134G), V256A (T767C)
	1R2	V108G (T323G), L240Q (T719A), V256A (T767C)
	1R3	(t120c), V256A (T767C), D275G (A824G)
	1R4	(c39t), N252D (A753G), V256A (T767C)
	1R5	D40E (G117A), (t339c), V256A (T767C)
2nd	2R1	1R4 mutations, (a216t), F90L (T270A), (t702c)
	2R2	1R4 mutations, (c243t), T195A (A585G)

* Substitutions of nucleotides are presented in parenthesis. Silent mutations are shown in lowercase letters.

### Effect of CBM6 Fusion on Activity of Thermotolerant Mutant Cel5A_2R2

**Figure 3:**
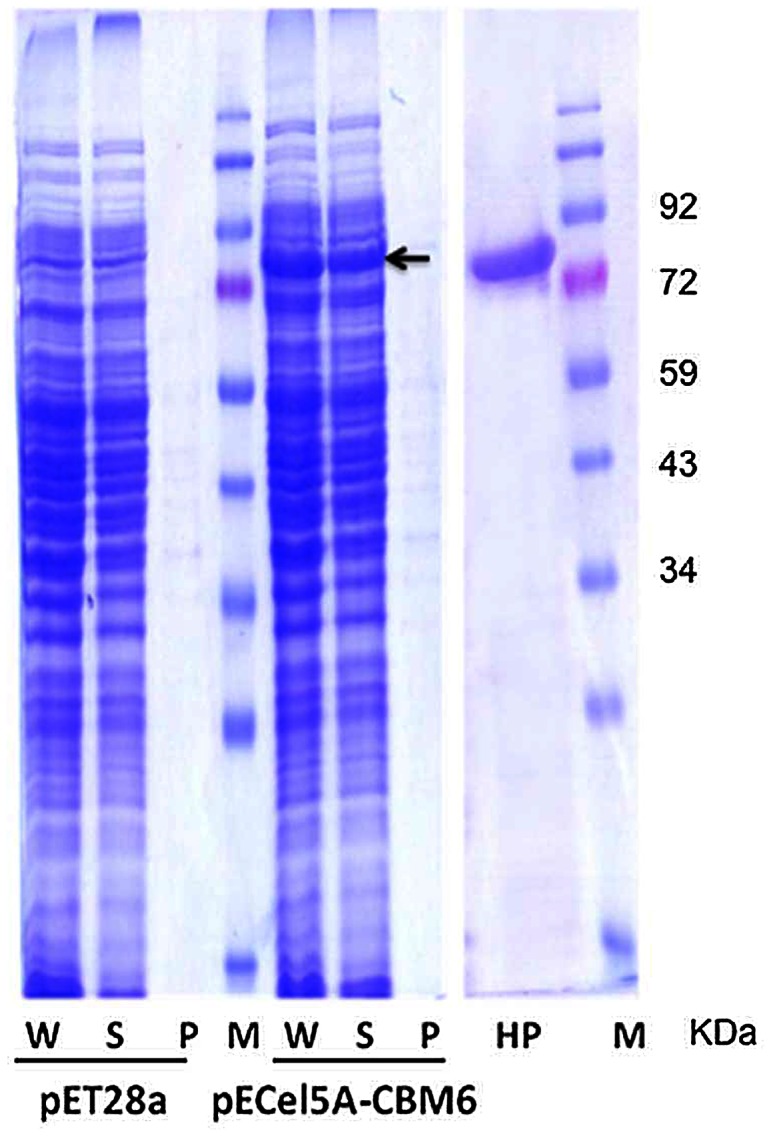
SDS-PAGE profile for expression and purification of Cel5A_2R2-CBM6 fusion protein. Lanes are as follows: W, whole cell proteins; S, soluble proteins; P, cell pellet proteins; HP, His-tag purified protein; and M, molecular weight marker.

The family-6 CBM from *S. degradans* was fused to the *C*-terminus of the thermotolerant mutant Cel5A_2R2 using the overlap PCR technique as described in Figure S1. The gene encoding the Cel5A_2R2-CBM6 fusion protein was subsequently inserted into the pET-28a(+) expression vector that contained an *N*-terminal His_6_ tag. The resulting construct was used for protein expression in *E. coli* BL21 (DE3). Expressed protein was extracted using CelLyticB reagent and soluble expression was evaluated using SDS-PAGE (Figure 3). The purified recombinant protein gave a single band on SDS-PAGE and its molecular size corresponded well with the theoretical molecular weight (72 kDa) calculated based on the amino acids sequence (Figure 3).

**Figure 4:**
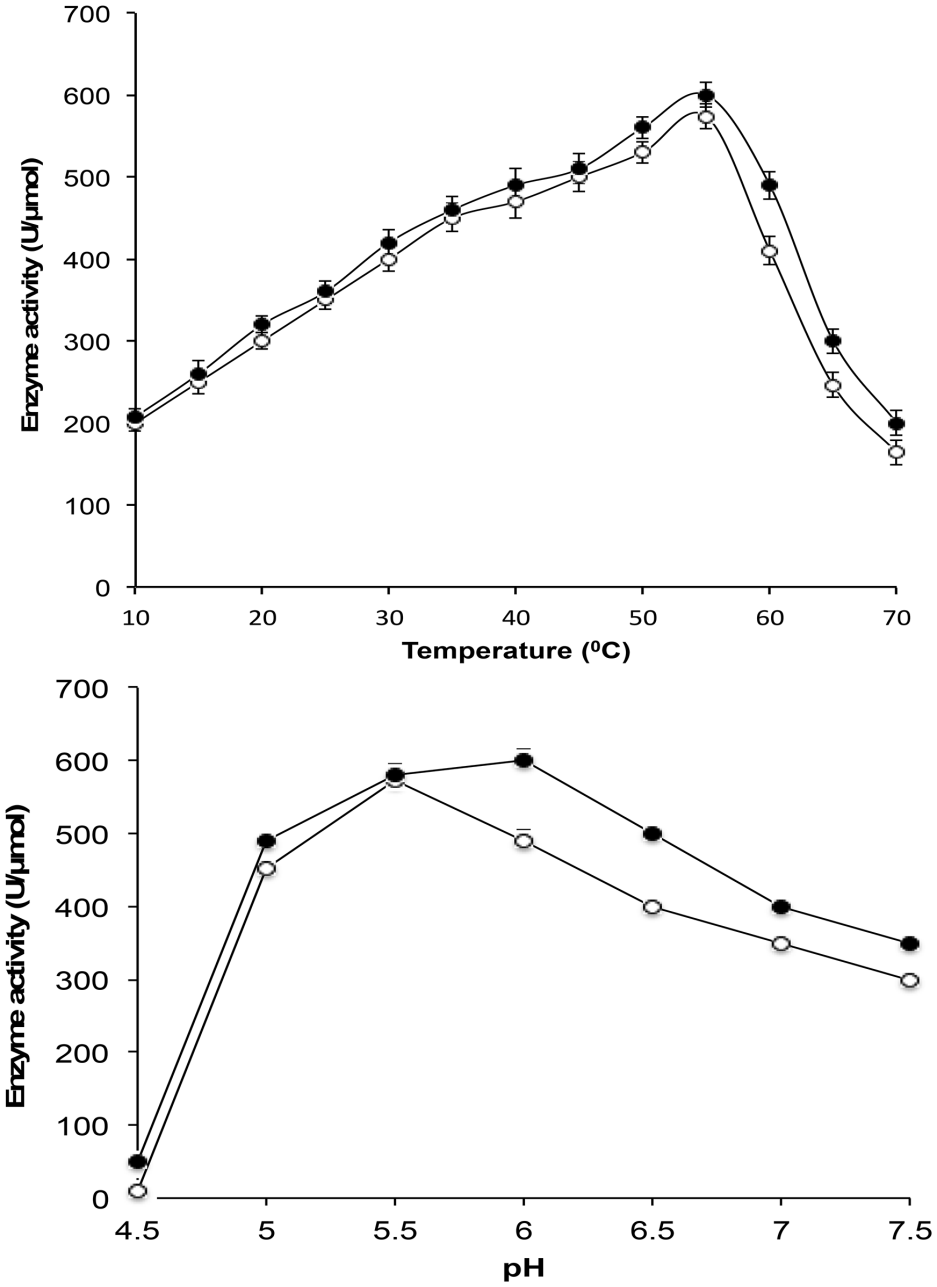
Optimum temperature and pH for Cel5A_2R2 and Cel5A_2R2-CBM6 fusion protein. Symbols are as follows: Cel5A_2R2 (Open circles) and Cel5A_2R2-CBM6 (Closed circles). The error bars represent the standard deviation of triplicate measurements.

The optimal temperature and pH of Cel5A_2R2-CBM6 fusion protein were investigated over a temperature range of 10–60°C and a pH range of 4.5–7.5 (Figure 4). The optimum pH shifted slightly to 6.0 in the fusion to CBM 6 compared with the optimum pH 5.5 of Cel5A_2R2, although there was no change in the optimum temperature as 55°C. Specific enzyme activity was also compared on different cellulosic substrates (Table 2). Cel5A_2R2 showed activity towards aryl glycoside (*p*-NPC) and soluble cellulose substrates (CMC, barley-β-glucan, and PASC) and a poor activity towards insoluble cellulose substrates such as filter paper and Avicel. Fusion with CBM6 did not affect the activity of the parent enzyme Cel5A_2R2 towards soluble cellulose substrates, but significantly improved activity towards insoluble cellulose substrates (Table 2). Catalytic activities of the fusion protein Cel5A_2R2-CBM6 towards filter paper and Avicel were increased about 7-fold compared to those of Cel5A_2R2. Avicel binding affinity of the Cel5A_2R2-CBM6 fusion protein was found to correlate with the enhanced catalytic activity on insoluble cellulosic substrates. Cel5A_2R2-CBM6 showed a higher binding affinity of 35% towards Avicel compared to 5% for Cel5A_2R2 (Figure S5).

**Table 2:** Specific enzyme activity of Cel5A_2R2 and Cel5A_2R2-CBM6 on various soluble and insoluble cellulosic substrates.

Substrates	Specific enzyme activity (U/µmol)		
	Cel5A	Cel5A_2R2	Cel5A_2R2-CBM6
	(41.6 kDa)	(41.6 kDa)	(72.0 kDa)
CMC	615±35	574±35	600±40
Barley β-glucan	3,490±90	3,410±100	3,480±100
PASC	80±10	75±10	144±10^a^
Filter paper	0.41±0.05	0.40±0.05	2.88±0.5^a^
Avicel	0.21±0.05	0.20±0.05	1.44±0.5^a^
*p-*NPC	98±10	95±10	90±10
*p-*NPG	ND^b^	ND^b^	ND^b^

a Specific activity of Cel5A_2R2-CBM6 was statistically significant from wild type Cel5A and mutant Cel5A_2R2 at two-tailed P value is less than 0.0001.

b ND represents “Not Detected”, indicating no enzyme activity.

### Hydrolysis Products of Cel5A_2R2-CBM6 Fusion Protein

**Figure 5:**
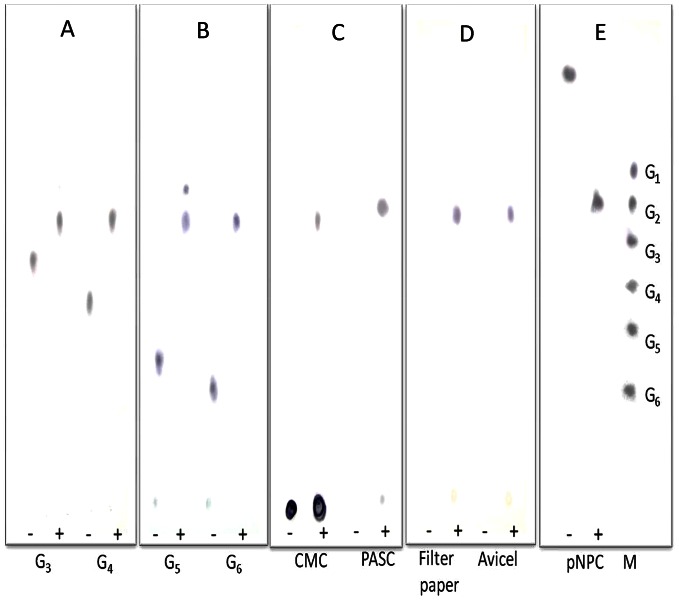
TLC analysis of hydrolysis products of cellotriose, cellotetraose, cellopentaose, cellohexaose, CMC, PASC, filter paper, Avicel, and *p*-NPC. A: Hydrolysis (1 h) products of cellotriose and cellotetraose, B: Hydrolysis (1 h) products of cellopentaose and cellohexaose, C: Hydrolysis (5 h) products of CMC and PASC, D: Hydrolysis (16 h) products of filter paper and Avicel, and E: Hydrolysis (1 h) product of *p*-NPC. M: Standard marker, where G1 to G6 represent glucose, cellobiose, cellotriose, cellotetraose, cellopentasoe, and cellohexaose. Cello-oligosaccharides, CMC, PASC, and *p*-NPC were treated with 0.1 nmol of Cel5A_2R2-CBM6 at 55°C. The same reaction was performed using Avicel and filter paper with 1.0 nmol of Cel5A_2R2-CBM6. Reactions were performed in the absence (−) and presence (+) of the enzyme.

Hydrolysis products of CMC, PASC, filter paper, and Avicel by the Cel5A_2R2-CBM6 fusion protein were qualitatively analyzed using TLC (Figure 5). Cellobiose was detected as a main product from the extensive hydrolysis of these polymeric cellulosic substrates. The Cel5A_2R2-CBM6 fusion protein cleaved cellotriose, cellotetraose, cellopentaose, and cellohexaose to produce cellobiose as a final product. A viscometric assay of CMC degradation was performed to investigate the reaction mode of the parent Cel5A_2R2 and the fusion Cel5A_2R2-CBM6 proteins. Viscosity of the CMC solution (1%, w/v) decreased after incubation with Cel5A_2R2 and Cel5A_2R2-CBM6 (Figure S6). A sharp decrease in the CMC viscosity indicated endoglucanase activity of the Cel5A_2R2 and Cel5A_2R2-CBM6 proteins as compared to cellobiohydrolase. These results suggest that Cel5A_2R2 and Cel5A_2R2-CBM6 possess endo-type activity and generally hydrolyze cellulose to cellobiose as a major product.

## Discussion

Directed evolution is a powerful technique for improving thermostability by generating random mutagenesis genetic libraries. Its prerequisite condition involves development of an effective and reliable screening method. We simultaneously applied two screening methods to identify desired mutants from an EP-PCR library. For easy and efficient screening of large libraries, we first used the Congo red plate assay to select clones with halo-forming activity that helped to remove numerous null mutants. The second screening procedure was to quantify enzyme activity at an elevated temperature, which helped us to identify thermotolerant mutants among the clones selected from the first screening stage. Our aim was to obtain and identify the best Cel5A mutant showing improved thermostability.

**Figure 6:**
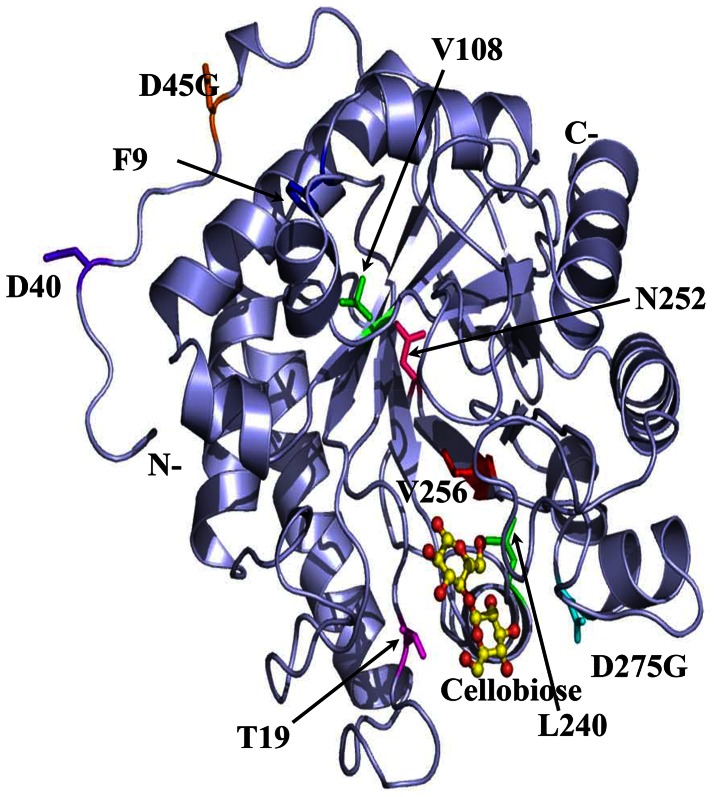
Cel5A mutations covered in this work mapped onto the model of the Cel5A catalytic domain. Cellobiose at the reaction cavity is displayed as ball-and-sticks (carbon in yellow and oxygen in red). Mutated residues in each mutant are shown as sticks in different colors: D45G in orange from 1R1, V108G, and L240Q in green from 1R2, D275G in cyan from 1R3, N252D in hot pink from 1R4, D40E in purple-blue from 1R5, T195A in magenta from 2R1, F90L in blue from 2R2, the common mutation V256A in red.

Protein engineering often faces a trade-off between improving desired properties and worsening others, such as thermal stability versus catalytic activity. This study focused on the screening and selection of mutants with increase of thermotolerance as well as minimization of catalytic activity loss. Although some decreases of specific enzyme activity and expression were observed for the mutants, the decreases were minimal. Several reports have showed that improvements in one cellulases property are independent of other properties, e.g., thermostabilization without a significant loss in catalytic activity [5], [27], [28]. The elevation of the optimum reaction temperature of Cel5A_2R1 and Cel5A_2R2 mutants seems to be related to the mutations effecting on Cel5A active site stabilization. The mutations were categorized to 4 groups according to their positions in the structure of Cel5A enzyme, mutations D45G of 1R1 and D40E of 1R5 in the *N*-terminal loop; N252D (α5/β6) of 1R4, F90L (α1) of 2R1, and T195A (β4/α4) of 2R2 in the loop regions inside the (α/β)8 TIM-barrel; V108G (β2) and L240Q (α6) of 1R2, V256A (β6) of 1R4, D275G (α6) of 1R3 in the barrel secondary structures; the F90L, V108G and G275G on the surface. Mutated amino acids were mapped and represented onto the crystal structure of Cel5A (PDB ID: 4HUO) (Figure 6). All mutants showed lower total energy (E_total_) than the wild type, suggesting that the mutants were more stable than the wild type (Table S4). Among the mutants, 1R3 showed the smallest ΔE (E_total, mutant_–E_total, WT_), 1R2 showed the largest ΔE, and the 2R1, and 2R2 mutants showed slightly higher total energy than 1R4, although these mutants also showed higher residual activity at 65°C. Other factors were also examined, which may affect thermostability, such as changes in solvent accessible surface area and length of the loops connecting secondary structure elements after the mutations [29], [30]. We calculated buried surface area of atoms in each mutant model (Table S4). Because changing only 2 or 3 amino acids does not significantly change relative surface area, we compared the change of total solvent accessible surface area (ΔA) between the wild type and each mutant. All mutants showed smaller surface areas than the wild-type enzyme, suggesting that the mutant protein may fold more compactly and have higher stability than the wild type. 1R1 showed the largest solvent accessible surface area change and 1R5 showed the smallest surface area change among the mutants (Table S4). Although no remarkable differences were observed near the catalytic site (electrostatic potential and surface area, data not shown), the mutations 1R4, 2R1, and 2R2 were distantly located from the catalytic site, and were more negatively charged near the region (Figure 6). Additionally, these electrostatic potential changes in the Cel5A mutants may optimize the full-length Cel5A-CBM6 conformation to increase the activity. The V256A mutation was common to all mutants. The V256A mutation may be essential for all gain-of-function mutants. V256A is located in the β6 strand below the substrate-binding site and is surrounded by side chains of polar and charged amino acids such as E189, H258, and T288. The region around V256 may be less stable because hydrophobic side chain of V256 is buried by polar residues. It appears that mutating V256 to alanine, a smaller hydrophobic side chain, makes the region more stable and compact. We compared amino acid sequences and structural arrangement around this area in our Cel5A with those of other 3 Cel5As from *Bacillus subtilis*, *Thermobifida fusca*, and *Thermotoga maritima* by using sequence alignment and by superimposition of the structures (Figure S7) using the Dali server [31]. The structures of the thermophillic family-5 cellulase from *B. subtilis* (PDB ID: 3PZV), *T. fusca* (PDB ID: 2CKS), and *T. maritima* (PDB code 3MMU) have been described previously [8], [32], and have similar polar and charged environments around the residue corresponding to V256 in our Cel5A. Interestingly, the family-5 cellulase from *B. subtilis* and *T. maritima* possess Ala and Thr at the residue corresponding to V256 in the wild-type enzyme, respectively. Packing of this area with the small hydrophobic residue alanine or a different small polar residue may play an essential role in enzyme thermostability. The mutations D40E, N252D and F90L, located on the loop surface, are conservative mutations. Thus, they may increase thermostability by reducing loop region flexibility. The residues D45, V108, and D275 are mutated to glycine residues. Glycine has broader conformational freedom than other amino acid residues, which may change local conformations around these residues and make this region more compact. The T195A, L240Q, and V256A mutations are located near the substrate binding and active site. The mutation L240Q behind V256A introduces additional hydrogen bonding interactions with the main chains of V256A and F276, presumably making the loop region more compact. The T195A mutation is located in the neighboring β strand close to the V256A residue and below the side chain of W236, which interacts with the bound sugar hexose ring. It appears that these 3 residue mutations, T195A, V256A, and L240Q, make the local structure around the active site more compact and stable, leading to higher thermostability of the enzyme.

Cel5A_2R2 showed good catalytic activity towards soluble cellulosic substrates, but low activity towards insoluble cellulosic substrates. We suspected that the absence of CBM in Cel5A might be responsible for its poor activity towards the insoluble cellulosic substrates of filter paper and avicel. Although Cel5A is an endoglucanase, it produces cellobiose as a major hydrolysis product from cellooligomers (G1 to G6) and polymeric cellulose substrates, including CMC and PASC (data not shown). A unique characteristic of processive endoglucanases is the production of cellobiose as a cleavage product, and their reaction rate for crystalline cellulose hydrolysis depends upon the non-catalytic CBM [25]. Thus, we fused CBM to the *C*-terminus of the thermotolerant mutant Cel5A_2R2 because its *N*-terminus possessed a secretion-signal sequence. Choosing a suitable CBM for processive endoglucanase activity is critical for generating an efficient fusion protein. To date, 64 CBM families have been described (http://www.cazy.org/). The cellulolytic mechanism of the Gram-negative bacterium *S. degradans* is well characterized and the family 5 endoglucanase (Cel5H) is reported to have a central role in cellulose degradation [20], [33]. The endoglucanase Cel5H has family 5 catalytic domains, and the family 6 CBM and has been extensively studied. *In vitro* studies have shown that artificially removing the family 6 CBM results in decreased endoglucanase Cel5H activity on filter paper and Avicel [20]. The CBM6 is separated from the catalytic domain by a polyserine linker (PSL). Previously, it was thought that the cellulase hydrolysis activity was also dependent upon the linker region, which joins the catalytic domain and CBM to each other. The use of CBM6, including the PSL, from Cel5H is reasonable for abolishing the efforts needed to design a new linker. Therefore, it was suspected that CBM6 and PSL from endoglucanase Cel5H were suitable choices for constructing the Cel5A_2R2-CBM6 fusion protein. The Cel5A_2R2-CBM6 fusion protein was successfully constructed, expressed, and purified to homogeneity. The catalytic efficiencies of cellulases were shown to be directly related to their substrate affinity [34], [35]. Cel5A_2R2-CBM6 fusion protein presented the significantly higher binding affinity and catalytic activity towards Avicel than the parent Cel5A_2R2.

**Figure 7:**
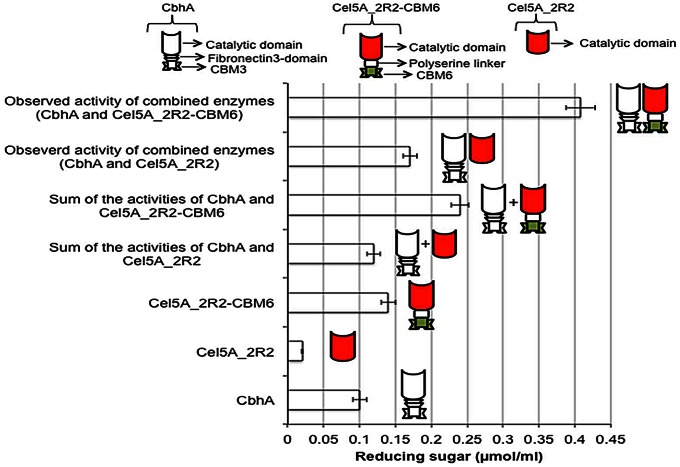
Synergistic interaction of cellobiohydrolase (CbhA) from *C. thermocellum* with Cel5A_2R2 parent protein and Cel5A_2R2-CBM6 fusion protein. The error bars represent the standard deviation of triplicate measurements.

The production of cellobiose by Cel5A_2R2-CBM6 may be an indication of enzyme processivity. Processive endoglucanase shows a good synergistic interaction with cellobiohydrolase [36]. We used cellobiohydrolase (CbhA) from the thermophilic bacterium *Clostridium thermocellum* to determine the synergetic effect between the fusion protein Cel5A_2R2-CBM6 and the parent Cel5A_2R2 (Figure 7). Total activity of the combined enzymes was greater than the sum of the individual enzyme activities. The Cel5A_2R2-CBM6 fusion protein showed a degree of synergistic effect (DSE) of 1.7 with CbhA for the degradation of filter paper, whereas the Cel5A_2R2 parent enzyme showed a low DSE of 1.2. This suggests that CbhA has a higher DSE with Cel5A_2R2-CBM6 than Cel5A_2R2. Complete cellulose hydrolysis is accomplished by the combined action of multiple enzymes such as cellobiohydrolase, endoglucanase, cellodextrinase, and β-glucosidase. This synergistic action among multiple enzymes is desired for effective cellulose hydrolysis. Therefore, the Cel5A_2R2-CBM6 fusion protein may be a candidate for developing cellulases mixture (cocktail) for complete hydrolysis of cellulosic substrates to fermentable sugars as compared to wild-type enzyme. We are currently conducting studies to investigate the synergetic effect of the Cel5A_2R2-CBM6 fusion protein with other cellobiohydrolases and endoglucanases to develop an effective cellulases cocktail.

### Conclusion

Random mutagenesis and CBM fusion protein approaches were successfully utilized to improve thermostability and catalytic activity of native Cel5A protein for cellulosic substrates, respectively. It was resulted into a thermotolerant engineered cellulase, Cel5A_2R2-CBM6. The Cel5A_2R2-CBM6 endoglucanase showed good synergy with cellobiohydrlase CbhA and is a candidate for developing a thermostable cellulase cocktail. This approach can be used to improve cellulolytic activities of wild-type cellulases.

## Supporting Information

Figure S1 **Schematic diagram for construction of Cel5A_2R2-CBM6 fusion protein.** The CBM6 from *S. degradans* is fused to C-terminal of Cel5A_2R2. The CD and PSL represent a catalytic domain and a polyserine linker, respectively.
(TIFF)

Figure S2 **Congo-red plate assay for visualization of Cel5A presence in extracellular site.** *E. coli* BL21 transformants (1×10^6^ CFU) harboring pTrc99A, pTw/o-ssCel5A (without a secretion signal sequence) and pTCel5A (with a secretion signal sequence) were spotted onto LB-ampicillin-agar plates and incubated at 37°C for 6 h and hydrolytic activity was checked by Congo red plate assay.
(TIFF)

Figure S3 **SDS-PAGE of periplasmic fractions from recombinant** ***E. coli*** **harboring pTrc99A, pTw/o-ssCel5A, and pTCel5A, respectively (lanes 1 to 3).** Lane M indicates molecular weight marker.
(TIFF)

Figure S4 **SDS-PAGE of purified wild-type Cel5A and its mutant proteins. 10 µg of purified protein was loaded in each lane.** Lanes 1 to 3 represent the purified proteins of wild-type Cel5A, mutant Cel5A_2R1, and mutant Cel5A_2R2. Lane M indicates molecular weight marker.
(TIFF)

Figure S5 **Binding affinity of Cel5A_2R2 and its CBM6 fusion protein, Cel5A_2R2-CBM6 to Avicel.** The error bars represent the standard deviation of triplicate measurements.
(TIFF)

Figure S6 **Viscosity profile of the CMC solution (1%, w/v) treated with Cel5A_2R2 (sqaure), Cel5A_2R2-CBM6 (circle), and cellobiohydrolase A (triangles).** The error bars represent the standard deviation of triplicate measurements.
(TIFF)

Figure S7 **Amino acid sequence alignment of Cel5A homologues.** Alignment was performed using the ClustalW2 (http://www.ebi.ac.uk/Tools/msa/clustalw2) and Gene Doc (http://WWW.nrbsc.org/gfx/genedoc) programs. The residues for conserved catalytic glutamates E193 and E289 are marked with ‘*’ sign. V256 is marked by arrow. The residues suggested around V256 are marked with ‘†’ sign. Dark and light shading indicate identical and similar amino acids, respectively.
(TIFF)

Table S1 **Strains, plasmids and genomic DNAs used in this study.**
(DOCX)

Table S2 **Primers used in this study.**
(DOCX)

Table S3 **Biochemical properties of wild-type Cel5A and its thermotolerant mutants.**
(DOCX)

Table S4 **Energy and surface area calculation of wild-type Cel5A and its thermotolerant mutants in correlation with their residual activity at 65°C.**
(DOCX)

## References

[1] Bras JL, Cartmell A, Carvalho AL, Verze G, Bayer EA, et al (2011) Structural insights into a unique cellulase fold and mechanism of cellulose hydrolysis. Proc Natl Acad Sci U S A 108: 5237–5242. 21393568 10.1073/pnas.1015006108 PMC3069175

[2] Lynd LR, Laser MS, Bransby D, Dale BE, Davison B, et al (2008) How biotech can transform biofuels. Nat Biotechnol 26: 169–172. 18259168 10.1038/nbt0208-169

[3] Zhang YH (2008) Reviving the carbohydrate economy via multi-product lignocellulose biorefineries. J Ind Microbiol Biotechnol 35: 367–375. 18180967 10.1007/s10295-007-0293-6

[4] Percival Zhang YH, Himmel ME, Mielenz JR (2006) Outlook for cellulase improvement: screening and selection strategies. Biotechnol Adv 24: 452–481. 16690241 10.1016/j.biotechadv.2006.03.003

[5] Liu W, Zhang XZ, Zhang Z, Zhang YH (2010) Engineering of Clostridium phytofermentans Endoglucanase Cel5A for improved thermostability. Applied and Environmental Microbiology 76: 4914–4917. 20511418 10.1128/AEM.00958-10 PMC2901741

[6] Wang Y, Tang R, Tao J, Wang X, Zheng B, et al (2012) Chimeric cellulase matrix for investigating intramolecular synergism between non-hydrolytic disruptive functions of carbohydrate-binding modules and catalytic hydrolysis. J Biol Chem 287: 29568–29578. 22778256 10.1074/jbc.M111.320358 PMC3436196

[7] Zhao J, Shi P, Huang H, Li Z, Yuan T, et al (2012) A novel thermoacidophilic and thermostable endo-beta-1,4-glucanase from Phialophora sp. G5: its thermostability influenced by a distinct beta-sheet and the carbohydrate-binding module. Appl Microbiol Biotechnol 95: 947–955. 22189866 10.1007/s00253-011-3807-0

[8] Santos CR, Paiva JH, Sforca ML, Neves JL, Navarro RZ, et al (2012) Dissecting structure-function-stability relationships of a thermostable GH5-CBM3 cellulase from Bacillus subtilis 168. Biochem J 441: 95–104. 21880019 10.1042/BJ20110869

[9] Wang XJ, Peng YJ, Zhang LQ, Li AN, Li DC (2012) Directed evolution and structural prediction of cellobiohydrolase II from the thermophilic fungus Chaetomium thermophilum. Appl Microbiol Biotechnol 95: 1469–1478. 22215071 10.1007/s00253-011-3799-9

[10] Telke AA, Ghatge SS, Kang SH, Thangapandian S, Lee KW, et al (2012) Construction and characterization of chimeric cellulases with enhanced catalytic activity towards insoluble cellulosic substrates. Bioresour Technol 112: 10–17. 22409983 10.1016/j.biortech.2012.02.066

[11] Yasir M, Khan H, Azam SS, Telke AA, Kim SW, et al. (2013) Cloning and functional characterization of endo-β-1,4-glucanase gene from metagenomic library of vermicompost. J Microbiol 51. DOI 10.1007/s12275-013-2697-5 (In press) 10.1007/s12275-013-2697-5 23812813

[12] Sambrook J, Russell DW (2001) Molecular cloning : a laboratory manual. Cold Spring Harbor, N.Y.: Cold Spring Harbor Laboratory Press.

[13] Bendtsen JD, Nielsen H, von Heijne G, Brunak S (2004) Improved prediction of signal peptides: SignalP 3.0. J Mol Biol 340: 783–795. 15223320 10.1016/j.jmb.2004.05.028

[14] Emsley P, Cowtan K (2004) Coot: model-building tools for molecular graphics. Acta Crystallogr D Biol Crystallogr 60: 2126–2132. 15572765 10.1107/S0907444904019158

[15] Brunger AT (2007) Version 1.2 of the Crystallography and NMR system. Nat Protoc 2: 2728–2733. 18007608 10.1038/nprot.2007.406

[16] Brunger AT, Adams PD, Clore GM, DeLano WL, Gros P, et al (1998) Crystallography & NMR system: A new software suite for macromolecular structure determination. Acta Crystallogr D Biol Crystallogr 54: 905–921. 9757107 10.1107/s0907444998003254

[17] Winn MD, Ballard CC, Cowtan KD, Dodson EJ, Emsley P, et al (2011) Overview of the CCP4 suite and current developments. Acta Crystallogr D Biol Crystallogr 67: 235–242. 21460441 10.1107/S0907444910045749 PMC3069738

[18] DeLano WL (2002) The PyMOL Molecular Graphics System. http://wwwpymolorg.

[19] Zhang Y, Chen S, Xu M, Cavaco-Paulo A, Wu J, et al (2010) Characterization of Thermobifida fusca cutinase-carbohydrate-binding module fusion proteins and their potential application in bioscouring. Appl Environ Microbiol 76: 6870–6876. 20729325 10.1128/AEM.00896-10 PMC2953015

[20] Watson BJ, Zhang H, Longmire AG, Moon YH, Hutcheson SW (2009) Processive endoglucanases mediate degradation of cellulose by Saccharophagus degradans. J Bacteriol 191: 5697–5705. 19617364 10.1128/JB.00481-09 PMC2737977

[21] Zhang YH, Cui J, Lynd LR, Kuang LR (2006) A transition from cellulose swelling to cellulose dissolution by o-phosphoric acid: evidence from enzymatic hydrolysis and supramolecular structure. Biomacromolecules 7: 644–648. 16471942 10.1021/bm050799c

[22] Wood TM (1971) The cellulase of Fusarium solani. Purification and specificity of the -(1–4)-glucanase and the -D-glucosidase components. Biochem J 121: 353–362. 5119766 10.1042/bj1210353 PMC1176581

[23] Ghose T (1987) Measurement of cellulase activities. Pure Appl Chem 59: 257–268.

[24] Eckert K, Zielinski F, Lo Leggio L, Schneider E (2002) Gene cloning, sequencing, and characterization of a family 9 endoglucanase (CelA) with an unusual pattern of activity from the thermoacidophile Alicyclobacillus acidocaldarius ATCC27009. Appl Microbiol Biotechnol 60: 428–436. 12466883 10.1007/s00253-002-1131-4

[25] Li Y, Irwin DC, Wilson DB (2007) Processivity, substrate binding, and mechanism of cellulose hydrolysis by Thermobifida fusca Cel9A. Appl Environ Microbiol 73: 3165–3172. 17369336 10.1128/AEM.02960-06 PMC1907127

[26] Kang MS, Kang IC, Kim SM, Lee HC, Oh JS (2007) Effect of Leuconostoc spp. on the formation of Streptococcus mutans biofilm. J Microbiol 45: 291–296. 17846581

[27] Anbar M, Lamed R, Bayer A (2010) Thermostability enhancement of Clostridium thermocellum Cellulosomal endoglucanase Cel8A by a single glycine substitution. Chem Cat Chem 2: 997–1003.

[28] Liang C, Fioroni M, Rodriguez-Ropero F, Xue Y, Schwaneberg U, et al (2011) Directed evolution of a thermophilic endoglucanase (Cel5A) into highly active Cel5A variants with an expanded temperature profile. Bioresour Technol 154: 46–53. 10.1016/j.jbiotec.2011.03.025 21501637

[29] Russell RJ, Hough DW, Danson MJ, Taylor GL (1994) The crystal structure of citrate synthase from the thermophilic archaeon, Thermoplasma acidophilum. Structure 2: 1157–1167. 7704526 10.1016/s0969-2126(94)00118-9

[30] Chan MK, Mukund S, Kletzin A, Adams MW, Rees DC (1995) Structure of a hyperthermophilic tungstopterin enzyme, aldehyde ferredoxin oxidoreductase. Science 267: 1463–1469. 7878465 10.1126/science.7878465

[31] Holm L, Rosenstrom P (2010) Dali server: conservation mapping in 3D. Nucleic Acids Res 38: W545–549. 20457744 10.1093/nar/gkq366 PMC2896194

[32] Pereira JH, Chen Z, McAndrew RP, Sapra R, Chhabra SR, et al (2010) Biochemical characterization and crystal structure of endoglucanase Cel5A from the hyperthermophilic Thermotoga maritima. J Struct Biol 172: 372–379. 20599513 10.1016/j.jsb.2010.06.018

[33] Taylor LE, 2nd, Henrissat B, Coutinho PM, Ekborg NA, Hutcheson SW, et al (2006) Complete cellulase system in the marine bacterium Saccharophagus degradans strain 2–40T. J Bacteriol 188: 3849–3861. 16707677 10.1128/JB.01348-05 PMC1482929

[34] Klyosov AA (1990) Trends in biochemistry and enzymology of cellulose degradation. Biochemistry 29: 10577–10585. 2271668 10.1021/bi00499a001

[35] Carrard G, Koivula A, Soderlund H, Beguin P (2000) Cellulose-binding domains promote hydrolysis of different sites on crystalline cellulose. Proc Natl Acad Sci U S A 97: 10342–10347. 10962023 10.1073/pnas.160216697 PMC27026

[36] Vazana Y, Morais S, Barak Y, Lamed R, Bayer EA (2010) Interplay between Clostridium thermocellum family 48 and family 9 cellulases in cellulosomal versus noncellulosomal states. Appl Environ Microbiol 76: 3236–3243. 20348303 10.1128/AEM.00009-10 PMC2869131

