# Supplementary figures and images for: Engineering of Family-5 Glycoside Hydrolase (Cel5A) from an Uncultured Bacterium for Efficient Hydrolysis of Cellulosic Substrates
Source: PLoS One. 2013 Jun 13;8(6):e65727. doi: 10.1371/journal.pone.0065727 (PMC3681849; doi:10.1371/journal.pone.0065727)

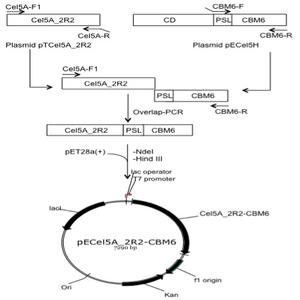

Supplement: Figure S1 — Schematic diagram for construction of Cel5A_2R2-CBM6 fusion protein. The CBM6 from S. degradans is fused to C-terminal of Cel5A_2R2. The CD and PSL represent a catalytic domain and a polyserine linker, respectively. (TIFF) [file pone.0065727.s001.tiff]

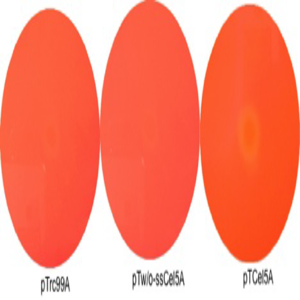

Supplement: Figure S2 — Congo-red plate assay for visualization of Cel5A presence in extracellular site. E. coli BL21 transformants (1×10^6^ CFU) harboring pTrc99A, pTw/o-ssCel5A (without a secretion signal sequence) and pTCel5A (with a secretion signal sequence) were spotted onto LB-ampicillin-agar plates and incubated at 37°C for 6 h and hydrolytic activity was checked by Congo red plate assay. (TIFF) [file pone.0065727.s002.tiff]

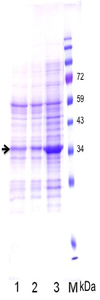

Supplement: Figure S3 — SDS-PAGE of periplasmic fractions from recombinant E. coli harboring pTrc99A, pTw/o-ssCel5A, and pTCel5A, respectively (lanes 1 to 3). Lane M indicates molecular weight marker. (TIFF) [file pone.0065727.s003.tiff]

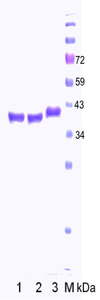

Supplement: Figure S4 — SDS-PAGE of purified wild-type Cel5A and its mutant proteins. 10 µg of purified protein was loaded in each lane. Lanes 1 to 3 represent the purified proteins of wild-type Cel5A, mutant Cel5A_2R1, and mutant Cel5A_2R2. Lane M indicates molecular weight marker. (TIFF) [file pone.0065727.s004.tiff]

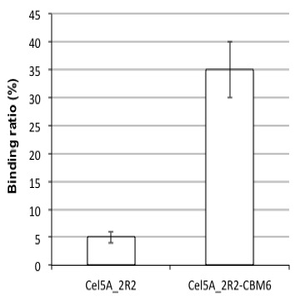

Supplement: Figure S5 — Binding affinity of Cel5A_2R2 and its CBM6 fusion protein, Cel5A_2R2-CBM6 to Avicel. The error bars represent the standard deviation of triplicate measurements. (TIFF) [file pone.0065727.s005.tiff]

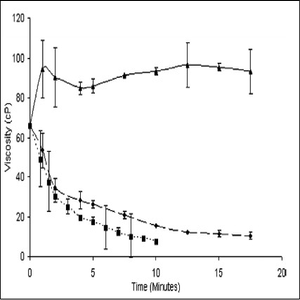

Supplement: Figure S6 — Viscosity profile of the CMC solution (1%, w/v) treated with Cel5A_2R2 (sqaure), Cel5A_2R2-CBM6 (circle), and cellobiohydrolase A (triangles). The error bars represent the standard deviation of triplicate measurements. (TIFF) [file pone.0065727.s006.tiff]

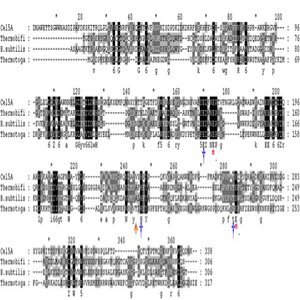

Supplement: Figure S7 — Amino acid sequence alignment of Cel5A homologues. Alignment was performed using the ClustalW2 (http://www.ebi.ac.uk/Tools/msa/clustalw2) and Gene Doc (http://WWW.nrbsc.org/gfx/genedoc) programs. The residues for conserved catalytic glutamates E193 and E289 are marked with ‘*’ sign. V256 is marked by arrow. The residues suggested around V256 are marked with ‘†’ sign. Dark and light shading indicate identical and similar amino acids, respectively. (TIFF) [file pone.0065727.s007.tiff]
