# Supplementary material for: Engineering of Family-5 Glycoside Hydrolase (Cel5A) from an Uncultured Bacterium for Efficient Hydrolysis of Cellulosic Substrates
Source: PLoS One. 2013 Jun 13;8(6):e65727. doi: 10.1371/journal.pone.0065727 (PMC3681849; doi:10.1371/journal.pone.0065727)
Supplement: Table S1 — Strains, plasmids and genomic DNAs used in this study. (DOCX) [file pone.0065727.s008.docx]

**Table S1**

| Strains, Plasmids, Genomic DNA | Descriptions | Sources |
| --- | --- | --- |
| **Strains** |  |  |
| *E. coli* DH5α | Gene cloning host | Invitrogen |
| *E. coli* BL21 | Protein expression host | Novagen |
| *E. coli* BL21(DE3) | Protein expression host | Novagen |
| *S. degradans* strain 2-40 | Source of processive endoglucanase Cel5H and family 6 CBM as a Gram negative cellulase producing bacterium | ATCC43961 |
| **Plasmids** |  |  |
| pTrc99A | Amp^r^, trc promotor, pBR322 ori | Novagen |
| pET28a(+) | Kan^r^, T7 promotor, pBR322 ori | Novagen |
| pTCel5A | pTrc99A vector containing wild type full length Cel5A gene. pTCel5A-Inner-F and Cel5A-R1 primers were used for Cel5Agene amplification. | This study |
| pTw/o-ssCel5A | pTrc99A vector containing a truncated Cel5A gene without signal sequence. Cel5A-F and Cel5A-R1 primers were used for the truncated w/o-ssCel5A gene amplification. | This study |
| pTCel5A-S-tag | pTrc99A vector containing a S-tag fused Cel5A gene. The pTCel5A-Inner-F and Cel5A-R2 were used for Cel5A-S-tag gene amplification. | This study |
| pTCel5A_1R1, 1R2, 1R3, 1R4, or 1R5 | pTrc99A vector containing Cel5A_1R1, 1R2, 1R3, 1R4, or 1R5 mutants. pTCel5A-Inner-F and Cel5A-R1 primers were used for mutant genes amplification. | This study |
| pTCel5A_2R1 | pTrc99A vector containing mutant Cel5A_2R1. pTCel5A-Inner-F and Cel5A-R1 primers were used for Cel5A_2R1 gene amplification. | This study |
| pTCel5A_2R2 | pTrc99A vector containing mutant Cel5A_2R2. pTCel5A-Inner-F and Cel5A-R1 primers were used for Cel5A_2R2 gene amplification. | This study |
| pECel5A | pET28a(+) vector containing a truncated Cel5A gene without signal sequence. Cel5A-F1 and Cel5A-R1 primers were used for the truncated Cel5A gene amplification. | This study |
| pECel5A_2R1 | pET28a(+) vector containing mutant a truncated Cel5A_2R1 without signal sequence. Cel5A-F1 and Cel5A-R1 primers were used for the truncated Cel5A_2R1 gene amplification. | This study |
| pECel5A_2R2 | pET28a(+) vector containing mutant a truncated Cel5A_2R2 without signal sequence. Cel5A-F1 and Cel5A-R1 primers were utilized for the truncated Cel5A_2R2 gene amplification. | This study |
| pECel5A_2R2-CBM6 | pET28a(+) vector containing Cel5A_2R2-CBM6 fusion gene. Cel5A-F1 and Cel5A-R1 primers were used for Cel5A_2R2-CBM6 gene amplification. | This study |
| pECbhA | pET28a(+) vector containing full length gene of cellobiohydrolase A. CbhA-F and CbhA-R primers were used for CbhA gene amplification. | This study |
| **Genomic DNAs** |  |  |
| *Clostridium thermocellum* | Genomic DNA as source of cellobiohydrolase A gene | ATCC 27405D-5 |
