# Supplementary material for: Engineering of Family-5 Glycoside Hydrolase (Cel5A) from an Uncultured Bacterium for Efficient Hydrolysis of Cellulosic Substrates
Source: PLoS One. 2013 Jun 13;8(6):e65727. doi: 10.1371/journal.pone.0065727 (PMC3681849; doi:10.1371/journal.pone.0065727)
Supplement: Table S2 — Primers used in this study. (DOCX) [file pone.0065727.s009.docx]

**Table S2**

| Primers | Sequences |
| --- | --- |
| pTCel5A-Outer-F | GCTGTTGACAATTAATCATCCGGCTCG |
| pTCel5A-Outer-R | CCGCCAGGCAAATTCTGTTTTATCAGACC |
| pTCel5A-Inner-F | ACAGGAAACAGACCATG**GAATTC** |
| pTCel5A-Inner-R | CATCCGCCAAAACAGCC**AAGCTT** |
| Cel5A-F | CATC**GAATTC**GACAACGCCTGGGAAACCAC |
| Cel5A-F1 | CTTG**CATATG**GACAACGCCTGGGAAACCAC |
| Cel5A-R | GGTGAGCCCTTATTGTCCTGCAACATC |
| Cel5A-R1 | GCTC**AAGCTT**ACTTATTGTCCTGCAACATC |
| Cel5A-R2 | CCC**AAGCTT**AGCTGTCCATGTGTTGGCGTTCAAATTT  CGCCGCCGCCGTTTCTTTCTACAACGTCCTGTTATTC |
| CBM6-F | CAGGACAATAAGGGCTCACCGAGCAGCAGCTC |
| CBM6-R | CGCTC**AAGCTT**ACCAGCTACCAAATTGCAGG |
| CbhA- F | CTA**GCTAGC**TATACTTCCGCAGCCTGAT |
| CbhA- R | TATAT**AAGCTT**AACCGCCCGGCGGCGTTCCCCA |

*Restriction enzyme sites are indicated in boldface. The overlapping PCR regions in fusion of CBM6 and Cel5A_2R2 are underlined.
