# Supplementary material for: Engineering of Family-5 Glycoside Hydrolase (Cel5A) from an Uncultured Bacterium for Efficient Hydrolysis of Cellulosic Substrates
Source: PLoS One. 2013 Jun 13;8(6):e65727. doi: 10.1371/journal.pone.0065727 (PMC3681849; doi:10.1371/journal.pone.0065727)
Supplement: Table S3 — Biochemical properties of wild-type Cel5A and its thermotolerant mutants. (DOCX) [file pone.0065727.s010.docx]

**Table S3**

| Properties | Cel5A | Cel5A_2R1 | Cel5A_ 2R2 |
| --- | --- | --- | --- |
| Expression level^a^ (μg/μl) | 2.0 | 1.8 | 1.5 |
| Optimum temperature (°C) | 50 | 55 | 55 |
| Optimum pH | 5.5 | 5.5 | 5.5 |
| Specific activity^b^ (U/µmol) | 615 ± 30 | 533 ± 40 | 574 ± 35 |

^a^Expression level was checked by S-tag rapid assay kit according to manufacturer’s instructions.

^b^Specific activity was determined using CMC as substrate.
