# Supplementary material for: Engineering of Family-5 Glycoside Hydrolase (Cel5A) from an Uncultured Bacterium for Efficient Hydrolysis of Cellulosic Substrates
Source: PLoS One. 2013 Jun 13;8(6):e65727. doi: 10.1371/journal.pone.0065727 (PMC3681849; doi:10.1371/journal.pone.0065727)
Supplement: Table S4 — Energy and surface area calculation of wild-type Cel5A and its thermotolerant mutants in correlation with their residual activity at 65°C. (DOCX) [file pone.0065727.s011.docx]

**Table S4**

| Model | WT | 1R1 | 1R2 | 1R3 | 1R4 | 1R5 | 2R1 | 2R2 |
| --- | --- | --- | --- | --- | --- | --- | --- | --- |
| Residual activity at 65°C | 10% | 17% | 20% | 26% | 40% | 30% | 50% | 60% |
| Etotal (Kcal/mol) | -14022.96 | -14484.35 | -14545.98 | -14206.03 | -14513.73 | -14530.97 | -14490.31 | -14511.64 |
| ΔE (Kcal/mol) | 0 | -461.39 | -523.02 | -183.07 | -490.77 | -508.01 | -467.35 | -488.68 |
| Surface area (Å2) | 13159.9 | 13094.3 | 13128.3 | 13124.0 | 13107.6 | 13132.4 | 13122.3 | 13114.3 |
| ΔS (Å2) | 0 | -65.6 | -31.6 | -35.9 | -52.3 | -27.5 | -37.6 | -45.6 |
